# Overexpression of sortilin is associated with 5‐FU resistance and poor prognosis in colorectal cancer

**DOI:** 10.1111/jcmm.15752

**Published:** 2020-12-16

**Authors:** Sabrina Blondy, Hugo Talbot, Sofiane Saada, Niki Christou, Serge Battu, Julie Pannequin, Marie‐Odile Jauberteau, Fabrice Lalloué, Mireille Verdier, Muriel Mathonnet, Aurélie Perraud

**Affiliations:** ^1^ Laboratoire EA3842 Contrôle de l’Activation Cellulaire Progression Tumorale et Résistances thérapeutiques «CAPTuR» Faculté de médecine Limoges France; ^2^ Service de Chirurgie Digestive Endocrinienne et Générale CHU de Limoges Limoges France; ^3^ IGF Université Montpellier CNRS INSERM Montpellier Cedex 5 France; ^4^ Service d’Immunologie CHU de Limoges Limoges France

**Keywords:** 5‐FU, CRC, resistance, sortilin

## Abstract

Colorectal cancer (CRC) is the third most common cancer worldwide. Even if 5‐fluorouracil (5‐FU) is used as the first‐line chemotherapeutic drug, responsiveness is only 20‐30%. Acquired resistance to 5‐FU contributes to both poor patient prognosis and relapse, emphasizing the need to identify biomarkers. Sortilin, a vacuolar protein sorting 10 protein (Vps10p), implicated in protein trafficking, is over expressed in CRC cell lines cultured 72 hours in presence of 5‐FU. This overexpression was also observed in 5‐FU‐resistant cells derived from these cell lines as well as in CRC primary cultures (or patients derived cell lines). A significantly higher expression of sortilin was observed in vivo, in 5‐FU‐treated tumours engrafted in Nude mice, as compared with non‐treated tumour. A study of transcriptional regulation allowed identifying a decrease in ATF3 expression, as an explanation of sortilin overexpression following 5‐FU treatment. In silico analysis revealed *SORT1* expression correlation with poor prognosis. Moreover, sortilin expression was found to be positively correlated with CRC tumour grades. Collectively, our findings identify sortilin as a potential biomarker of 5‐FU resistance associated with poor clinical outcomes and aggressiveness in CRC. As a new prognostic factor, sortilin expression could be used to fight against CRC.

## 1 INTRODUCTION

Worldwide, colorectal cancer (CRC) is the third most commonly occurring cancer and the second leading causes of cancer‐related deaths.^1^, ^2^ Systemic 5‐fluorouracil (5‐FU) has been used for decades, and remains the first‐line molecule used in CRC chemotherapy.^3^ It is currently used in combination with other antitumour agents such as irinotecan, oxaliplatin, folinic acid, and also targeted therapies. Unfortunately, 5‐FU resistance renders combined chemotherapies mostly ineffective^4^, ^5^ and leads to relapse and poor patient prognosis. Advanced tumour grade is associated with poor overall survival and disease‐free survival (DFS) after 5‐FU‐based systemic chemotherapy.^6^ The mechanisms involved in 5‐FU resistance are numerous and complex.^7^ These mechanisms remain at the centre of intensive research programmes to find the origin of the resistance^8^ or to discover new molecules that are able to overcome this resistance.^9^

Sortilin, which is ubiquitously expressed in mammalian cells,^10^ acts as a regulator of intra‐ and extra‐cellular protein sorting and trafficking,^11^, ^12^ as well as a co‐receptor of neurotrophins (NTs).^13^ The neurotrophin family consists of four members: nerve growth factor (NGF), brain‐derived neurotrophic factor (BDNF), NT4/5 and NT3. Each one of the four NTs strongly binds to a tropomyosin receptor kinase (Trk) receptor (NGF to TrkA, BDNF and NT4/5 to TrkB, and NT3 to TrkC) and promotes cell survival. All NTs also bind with low affinity to the neurotrophin receptor p75 (p75^NTR^) alone, then triggering cell death. NTs may also bind with higher affinity to the p75^NTR^/sortilin/Trk or sortilin/Trk complexes, and then they promote cell survival.^13^, ^14^, ^15^ The NTs pathways have been implicated in multiple types of cancer, notably digestive.^16^ TrkB/BDNF is the only NTs complex which has been firmly shown to play oncogenic roles in CRC development, including tumour growth, progression and cell survival.^17^, ^18^, ^19^, ^20^

The roles played by sortilin in cancers are more complex: depending on the origin of the tumour, it has been described either as a good or a bad prognosis marker of tumour aggressiveness. In breast cancer, ovarian carcinoma and in neuroendocrine tumours, targeting sortilin has been reported to inhibit tumour metastasis and to promote tumour cell apoptosis; this observation underscores the bad prognosis linked to a high level of sortilin expression.^21^, ^22^, ^23^, ^24^ On the opposite, our team has recently reported that the loss of sortilin promoted lung cancer cell proliferation, in relation with the epidermal growth factor receptor (EGFR) signalling efficiency.^12^ More especially, sortilin acts as a key regulator in balancing CRC cell survival and death through its association with, respectively, TrkB and p75^NTR^.^20^ Moreover, besides the role played by the full‐length protein, sortilin can also be cleaved and released as a soluble form that promotes the survival of cancer cells and enhances their invasive potential.^25^, ^26^ Therefore, sortilin can be considered as a multifunctional protein, able to play various roles, as a receptor, a trafficking chaperone or a soluble signalling molecule. A link between altered *SORT1* expression and cancer treatment has only been reported for B acute lymphoblastic leukaemia.^27^ However, to date, a relationship between sortilin expression and chemotherapy efficiency and, more specially, its role in 5‐FU resistance, has not been demonstrated yet.

This work was aimed at exploring a possible link between cancer therapy and sortilin expression in 5‐FU‐treated CRC cells. It was found that short‐ (72 hours) and long‐term (one month) treatments resulted in sortilin overexpression. This enhancement was evidenced in CRC cell lines, as well as in primary cultures and even in tumours obtained after transplantation of CRC cells in Nude mice. We also found that sortilin enhancement was accompanied with a down‐regulation of ATF3 expression. In silico analyses revealed that *SORT1* expression was associated with poor clinical outcomes. Moreover, we found that sortilin overexpression was associated with higher CRC tumour grades in patient tissue samples.

Our findings suggest that sortilin could be considered as a biomarker of 5‐FU resistance. Associated with other prognosis factors, for example, tumour grade, assessment of sortilin level might provide additional information about tumour aggressiveness, by increasing the accuracy of patient classification and could help to make treatment decisions.

## 2 MATERIALS AND METHODS

### 2.1 Reagents

Two human CRC cell lines, WiDr (well‐differentiated adenocarcinoma, stage Dukes C, low grade, microsatellite stable [MSS], v‐Ki‐ras2 Kirsten rat sarcoma viral oncogene homolog (KRAS)^WT^, v‐raf murine sarcoma viral oncogene homolog B1 (BRAF)^V600E^, phosphoinositide 3‐kinase (PI3KCA)^P449T^, phosphatase and tensin homolog (PTEN)^WT^) and SW620 (undifferentiated node metastasis, Stage Dukes C, high grade, MSS, KRAS^G12V^, BRAF^WT^, PI3KCA^WT^, PTEN^WT^)^28^, ^29^ were obtained from American Type Culture Collection (ATCC/LGC Promochem, Molsheim, France). Six primary cultures from patients suffering from different stages and grades of CRC, receiving adjuvant 5‐FU‐based chemotherapies, were obtained from IGF (Montpellier France), after informed consent of patients (Material Transfer Agreement CNRS 190287; Table 1). All cells were regularly tested for mycoplasma contamination.

**TABLE 1:** Clinical data of primary cultures

	(1)	(2)	(3)	(4)	(5)	(6)
Sex	M	F	M	M	M	F
Age	71	65	65	81	81	66
Cell location	P	P	Meta	P	Meta	P
Colic location	Sigmoïd	Left colon	Right colon	Transverse colon	Sigmoïd	Right colon
Synchronous metastases	yes	no	yes	no	yes	yes
Metastases location	Pulmonary	‐	Hepatic	‐	Hepatic	Hepatic
*KRAS* mutation	no	yes	yes	no	yes	yes
Tumour grade	Int	low	low	high	Int	Int
TNM	T4N2M1	NI	TXN + M1	T4aN0M0	TXNXM1	TXNXM1
TNM chimio	‐	‐	BEVATUXIMAB			
FOLFIRI	‐	‐	Avastin FOLIRI			
Radiotherapy	no	no	no	no	no	no
Chemotherapy	yes, but no information	no	Bevacizumab Folfiri	no	Bevacizumab Folfiri	no

Note

Six primary cultures were obtained from IGF Montpellier France, after informed consent of patients (Material Transfer Agreement CNRS 190287). At the time of cell recovery, patients were suffering from different tumour grades, pTNM stages (TNM and TNM chimio), and were treated as mentioned above with chemotherapies.

Abbreviations: F, female; Int, intermediate; M, male; Meta, metastases; P, primary tumour.

For Western blot analysis, primary antibodies used were mouse anti‐actin (A5441, Sigma‐Aldrich), mouse anti‐ATF3 (MA5‐31635, Thermofisher), rabbit anti‐p75^NTR^ (sc8317, Santa Cruz), mouse anti‐sortilin (612101, BD Biosciences), mouse anti‐AKT (#2920), rabbit anti‐ERK (#4695), rabbit anti‐PARP (#9542), rabbit anti‐P‐AKT (#4060), rabbit anti‐P‐ERK (#4370), rabbit anti‐α tubulin (#2144) from Cell Signaling Technology and rabbit anti‐TrkB (orb214339, Biorbyt).

For immunohistochemistry analysis, primary antibody used was mouse anti‐sortilin (ANT‐016, Alomone Labs).

For indirect immunofluorescence staining analysis, primary antibody was rabbit anti‐GM130 (PA1‐077, Thermofisher) and mouse anti‐sortilin (612101, BD Biosciences).

### 2.2 Cell culture and pharmacological treatments

CRC cell lines, WiDr and SW620, were cultured as previously described.^20^ The primary cultures were cultured in Dulbecco's modified eagle medium (DMEM) GlutaMAX (supplemented with 10% foetal bovine serum (FBS) and 100 IU/mL penicillin + 100 mg/mL streptomycin (Thermo Fisher, France).

To obtain long‐term 5‐FU‐resistant cells (one month), a modified protocol was followed.^30^ In,^30^ it took nearby 32 weeks to obtain resistant cells at a final 5‐FU concentration of 2µg/ml. Since the final concentration of 1 µg/mL, corresponding to the IC50 of the cell lines, was chosen, our revisited protocol allowed obtaining resistant cells in only 4 weeks. Cells were seeded in T75 flasks at 5 × 10^6^ cells/10 mL of media (WiDr) or 2.5 × 10^6^ cells/10 mL of media (SW620 and primary cultures) for untreated conditions, and 7 × 10^6^ (WiDr) or 5 × 10^6^ (SW620 and primary cultures) for 5‐FU treatment. During one month, 2 mL of media containing 5‐FU (Sigma‐Aldrich) was added every 72 hours to the cultures in order to reach a final concentration of 8 µmol/L, corresponding to IC50.

### 2.3 Nude mice xenografts and pharmacological treatment

Animal study was conducted in accordance with guidelines established by the regional Institutional Animal Care and Use Committee (Comité Régional d’Éthique sur l’Expérimentation Animale du Limousin n°87‐005). The same committee also approved the experimental protocol. Ten seven‐week‐old female NMRI Nude mice (Janvier Labs, St. Berthevin, France) were subcutaneously injected with 4 × 10^6^ WiDr or 2 × 10^6^ SW620 cells. Every 3 days, length (L) and width (W) were measured to determine tumour volume (*V* = [L × W(L + W)]·π/12). Once tumour volume reached 100 mm^3^ (day 0), mice were divided into two groups, untreated and treated (*n* = 3 mice per group; dead or unsuccessfully xenografted mice were excluded). Every 3 days for 21 days, mice from the untreated group (CTL) received intraperitoneal injection of 6% dimethyl sulfoxide (DMSO; Sigma‐Aldrich), and mice from the 5‐FU‐treated group (5‐FU) received 40 mg/kg 5‐FU diluted in 6% DMSO.^31^ At the end of treatment, animals were sacrificed and tumours were recovered for Western blotting and histological analyses.

### 2.4 Cellular metabolic activity

Cells were seeded in 96‐well plates. The next day, they were exposed to increasing doses of 5‐FU (0, 4, 8, 16, 32 and 64 µmol/L). Seventy‐two hours later, metabolic activity was determined with the thiazolyl blue tetrazolium blue (MTT) assay (CellTiter 96 Aqueous One solution Cell Proliferation Assay, Promega, Charbonnières‐les‐Bains, France) following supplier instructions. Results were expressed as percentage of the value obtained with untreated cells (defined as 100%).

### 2.5 Cell death and viability

Cell death was evaluated by flow cytometry using PI/Annexin V–fluorescein isothiocyanate (FITC) double staining, as previously described^32^ on a FACS Calibur flow cytometer (Becton Dickinson, Heidelberg, Germany). Calibration was performed using untreated cells (control) as reference.

### 2.6 Cell proliferative activity

After 5‐FU treatments, activated cells were determined by Cell Proliferation ELISA BrdU, colorimetric (Roche, Sigma‐Aldrich) assay. This test is based on the measurement of BrdU incorporation during DNA synthesis in proliferating cells. Untreated cells were used as control (100%).

### 2.7 Indirect immunofluorescence staining

Fixation, permeabilization/saturation and staining were performed as previously described.^20^ Cells were then incubated for 2 hours at room temperature with Alexa Fluor 488 or 594 nm conjugated secondary antibodies (Invitrogen) diluted 1:2500 in phosphate‐buffered saline (PBS); nuclei were then stained with 4',6‐diamidino‐2‐phenylindole (DAPI) for 5 min. Coverslips were mounted in Fluorescence Mounting Medium (Dako Cytomation) and observed by confocal microscopy with a 40× immersion objective using FITC and DAPI lasers (LSM 510 META; Zeiss, Göttingen, Germany). Images were analysed using Zeiss ZEN and ImageJ softwares (NIH, Bethesda, MD, USA).

### 2.8 Immunohistochemistry staining

Staining of sortilin was performed on tissue microarrays (TMA) of 133 patients (GTX21418 and GTX21433, Clinisciences) and paraffin‐embedded xenografted Nude mice tumours, as previously described.^12^, ^19^ Images were acquired with the Nanozoomer Digital Pathology 2.ORS software. Immunohistochemistry intensity scores were obtained by multiplying the score for staining intensity (0: no staining; 1: weak; 2: moderate; 3: high) with the score for the percentage of positive cells (0: no cells; 1:0‐10%; 2:11‐50%; 3:51‐100%).

### 2.9 Western blot

Proteins were extracted from whole‐cell lysates (15‐30 µg) and mouse tumour samples (50 µg) as previously described.^20^, ^33^ Subcellular fractionation was effected according to supplier instructions (Thermo Fisher, France). Proteins were blotted onto polyvinylidene fluoride membrane (Calbiochem/Merck Millipore) and incubated with primary antibodies. After washing in Tris‐buffered saline/0.1% Tween‐20, immunoreactions were detected as previously described^34^ using GeneSnap and GeneTool (Syngene, Cambridge, UK). Densitometric analyses were performed using the ImageJ software.

### 2.10 Real‐time quantitative polymerase chain reaction (RT‐q‐PCR)

RNA extraction, reverse transcription and real‐time quantitative PCR analyses were performed as previously described.^35^ Primers and probe sequences (Life Technologies) were as follows: *Hypoxanthine Phosphoribosyltransferase 1* (*HPRT1)* (Control mix, Applied Biosystems, TaqMan, VIC Catalog number: 4326321E); *SORT1* Forward (CTGGGTTTGGCACAATCTTT) and reverse (CACCTTCCTCCTTGGTCAAA). Relative mRNA levels were determined by normalization to *HPRT* under the reference condition (control defined as 1).

### 2.11 In silico analysis

*SORT1* expression levels by risk group in resected patients suffering from CRC and treated with chemotherapies, according to survival (Sveen Colon ‐ GSE24550),^36^, ^37^ DFS (Colon Metabase Uniformized dataset ‐ GSE12945), and RFS (Thorsteinsson Olsen Colon ‐ GSE31595)^38^ were extracted from SurvExpress‐Biomarker validation for cancer gene expression.

### 2.12 Statistical analysis

Results were analysed by Kruskal‐Wallis test for immunohistochemistry, and by two‐way ANOVA/ANCOVA for others (StatView 5.0 software; Abacus Concepts, Piscataway, NJ, USA; *P < .05, **P < .01, ***P < .001). Mean and SEM values were obtained from at least three independent experiments; variance being similar between the statistically compared groups. Neither randomization nor blinding was done.

## 3 RESULTS

### 3.1 A 72 hours 5‐FU treatment correlates with sortilin overexpression in CRC cell lines

**FIGURE 1:**
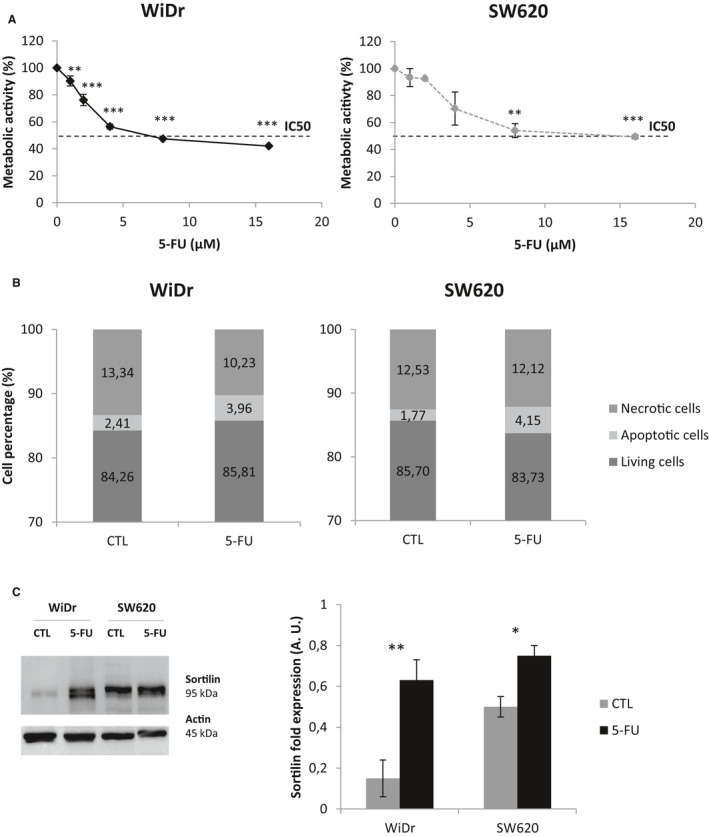
Reponses to 5‐FU short‐term treatment (8 µmol/L, 72 h) of WiDr and SW620: metabolic activity, cell death and sortilin expression. WiDr and SW620 were treated with 5‐FU for 72 h. (A, Increased 5‐FU concentrations were tested for metabolic activity analysis using MTT assay (Promega, France). Untreated cells were used as control (100%). B, Cell death induction after 5‐FU treatment (8 µmol/L, 72 h) was determined by flow cytometry with PI/AnnexinV‐FITC. C, Sortlin protein expression was analysed by Western blotting from whole‐cell lysates of 5‐FU‐treated WiDr and SW620 (8 µmol/L, 72 h) and ‐untreated cells (CTL). Actin was used as loading control. Histograms are the means from at least three independent experiments. Significant P‐values are indicated in the graphs *P < .05, **P < .01, ***P < .001

In previous work, we demonstrated that NTs are survival factors.^19^, ^20^ Since 5‐FU responsiveness is only 20‐30%, the possible link between NTs and 5‐FU treatment efficiency was studied in two different CRC cell lines. First, the appropriate 5‐FU concentration for treatment of WiDr and SW620 was determined. WiDr and SW620 were treated with increasing 5‐FU concentrations for 72 hours. As shown in Figure 1A, metabolic activity of the two cell lines decreased with increasing 5‐FU concentrations. A concentration of 8 µmol/L inhibited the metabolic activity of WiDr by 50% (IC50); SW620 cells were less sensitive, displayed 53.9% of their metabolic activity in the same conditions. Because significant difference with control cells was obtained with only 8 µmol/L for SW620, this experimental condition was chosen for further experiments. Moreover, we checked the blockade in the S cell cycle phase for this experimental condition ((*P* = 0;0003 for WiDr and *P* = 0,0006 for SW620; Figure 1SA).

As expected, even if apoptotic cells were detected after 5‐FU treatment, living cells represented more than 80% of the population of each one of the cell lines (85,8% for WiDr and 83,7% for SW620; Figure 1B). The difference in 5‐FU sensitivity could be explained by the impact on cell proliferation. Indeed, the proliferation rate of WiDr and SW620 cells dropped by factors of 2.3 and 1.9, respectively, so WiDr presented half the proliferation rate of SW620 (Figure 1SB). These data showed that 72 hours of 5‐FU treatment has a cytostatic effect on the cell lines, WiDr cells being more sensitive than SW620.

In a previous study, we demonstrated the implication of NTs in the survival of CRC cells.^19^, ^20^ So, even if we did not see any lethal effect of 5‐FU, we resolved to look after TrkB, p75^NTR^ and sortilin expression. After 72 hours of 5‐FU treatment, we did not observe any effect on TrkB and p75^NTR^ expression (Figure 1SC). However, a significant enhancement of sortilin expression was observed in both cell lines (Figure 1C; *P* = .007 for WiDr and *P* = .036 for SW620). By examining the main proliferative pathways linked to NTs signalling, the P‐Akt/Akt ratio increased in WiDr (*P* = 0,016), and significant changes were observed in SW620 (Figure S1D) with a decrease of the P‐Akt/Akt ratio (*P* = .004) and an increase of the P‐Erk/Erk ratio (*P* = .0003). However, because the P‐Erk/Erk signalling pathway seemed to increase in the two cell lines after 5‐FU treatment, and more especially in SW620, this result could justify the persistence of the proliferative activity of this latter cell line (Figure 1SB).

### 3.2 Long‐term (one month) 5‐FU‐resistant cells exhibit sortilin overexpression

**FIGURE 2:**
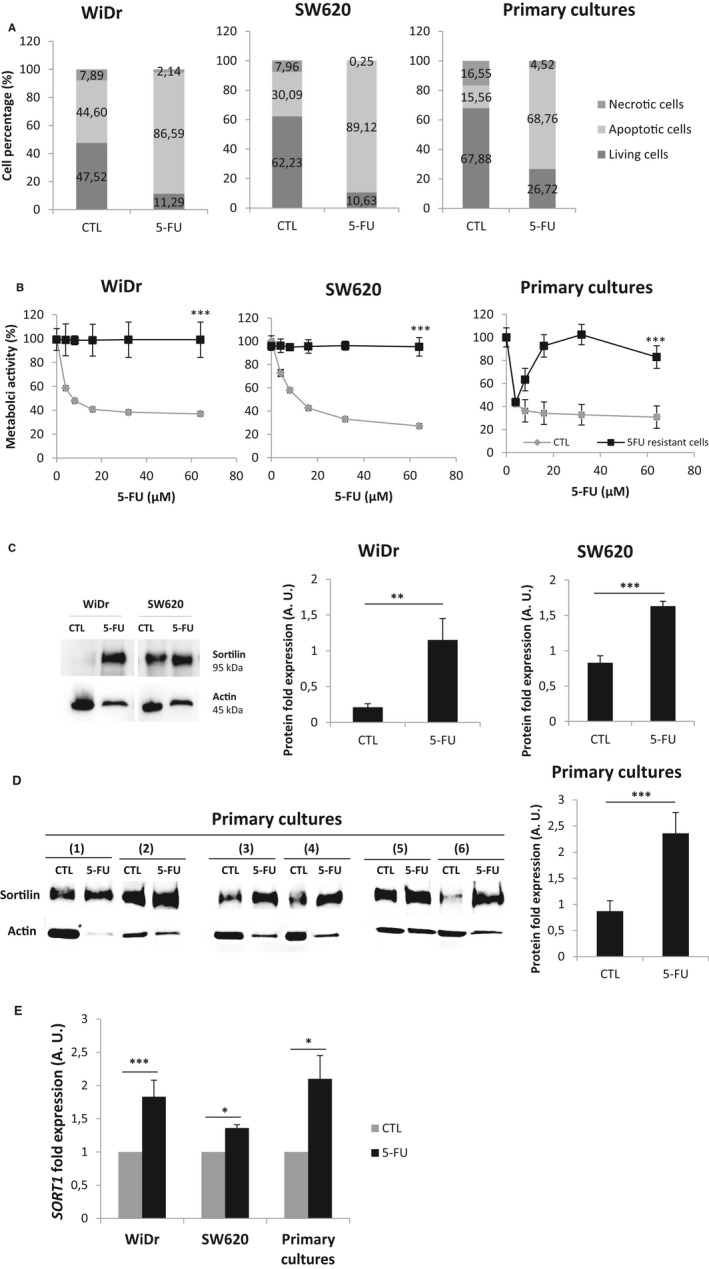
Reponses to 5‐FU long‐term treatment (8 µmol/L, one month) of WiDr, SW620 and primary cultures: cell death, insensitivity to 5‐FU increasing concentrations and sortilin expression. After one month of 5‐FU treatment, A, cells were analysed by flow cytometry for detection of living and dead cells (PI/Annexin‐FITC) as described before. Histograms are means from at least three independent experiments. B, Metabolic activity of 5‐FU‐resistant cells was determined using MTT assay (Promega, France). Untreated cells (maintained in culture during one month) were also analysed and used as control (CTL). Significant P‐values are indicated in the graphs: *P < .05; **P < .01; ***P < .001. Sortlin protein expression was analysed by Western blotting from whole‐cell lysates of 5‐FU‐resistant (5‐FU) and ‐untreated cells (CTL) in WiDr and SW620 C, and in primary cultures D,. Actin was used as loading control. Histograms are the means from at least three independent experiments. Significant P‐values are indicated in the graphs *P < .05, **P < .01, ***P < .001. E, Sortilin transcriptomic expression of 5‐FU‐resistant cells (one month) and ‐untreated cells (CTL) was analysed by RT‐q‐PCR. Sortilin mRNA expression (*SORT1*) upon 5‐FU was normalized to both *HPRT* transcriptomic expression and that of untreated cells. Histograms are the means from at least three independent experiments. Significant P‐values are indicated in the graphs *P < .05, **P < .01, ***P < .001

This short‐term 5‐FU treatment (72 hours) had cytostatic rather than cytotoxic effects, since the percentage of apoptotic cells failed to vary between control and treated conditions in both cell lines (Figure 1B). So, we resolved to extend the 5‐FU exposure time to one month (8 µmol/L every 72 hours) based on IC50 for WiDr, SW620 and six primary cultures. Even if this prolonged 5‐FU treatment induced an important drop in living cells (85% for WiDr, 88% for SW620 and 72% for primary cultures), it allowed the survival of more than 10% of the cells (Figure 2A). At the end of the treatment, surviving cells were exposed to increasing doses of drug (0 to 64 µmol/L) for 72 hours and were shown do display a strong resistance to 5‐FU. Metabolic activity of these 5‐FU‐resistant cells showed absolutely no sensitivity towards 5‐FU, up to 64 µmol/L, contrary to control cells which had been maintained in drug‐free culture during one month (Figure 2B). This acquired resistance was obtained with the two cell lines and with primary cultures.

As before, we evaluated NTs receptor (TrkB, p75^NTR^ and sortilin) expression in 5‐FU‐resistant cell issued from the two lines and from primary cultures. Once again, after one month of 5‐FU treatment, only the expression of sortilin was significantly up‐regulated in each cell line (*P* = .008 for WiDr and *P* = .004 for SW620; Figure 2C) and in primary cultures as well (*P* = .002; Figure 2D). Similarly to the data obtained after 72 hours treatments, NTs receptor expression was unchanged (data not shown). Probably due to the low proportion of living cells, proliferative pathways were dramatically affected and were not exploitable (data not shown). Sortilin mRNA levels were also higher in all 5‐FU‐resistant in comparison with control cells (*P* = .004 for WiDr, *P* = .013 for SW620 and *P* = .027 for primary cultures; Figure 2E), suggesting that *SORT1* was transcriptionally up‐regulated. Regardless of tumour stage and cellular origin (cell lines or primary cultures), sortilin expression was significantly elevated at both the protein and mRNA levels in 5‐FU‐resistant cells. Consistent with observations obtained with a 72 hours treatment, sortilin seems to behave as a biomarker of 5‐FU resistance in CRC regardless of the duration of the treatment and mutational status as well.

### 3.3 5‐FU disrupts sortilin localization and transcriptional regulation

**FIGURE 3:**
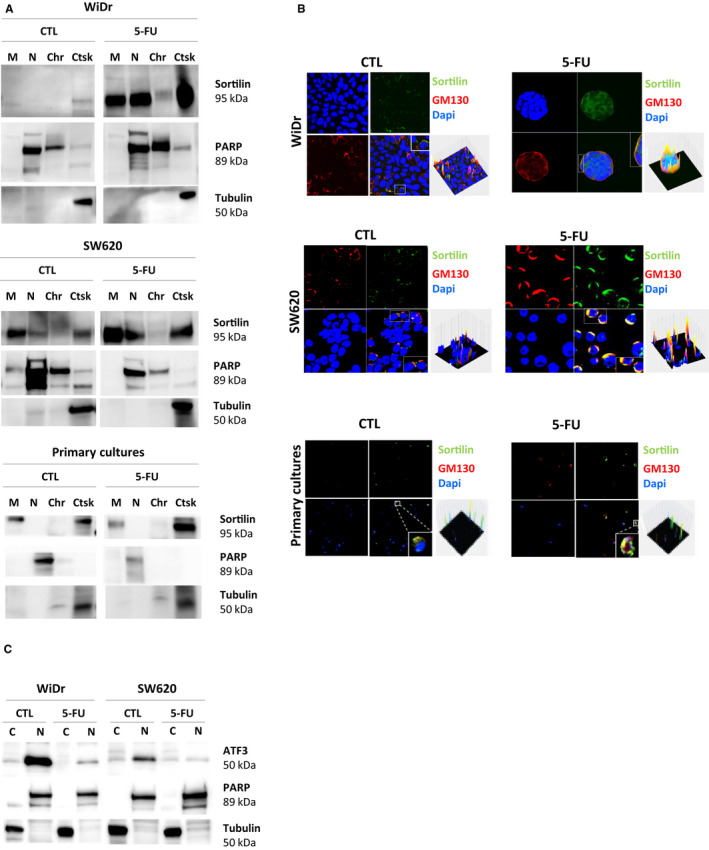
Sortilin localization and transcriptional regulation upon long‐term 5‐FU treatment. A, To analyse sortilin localization, a subcellular fractionation was done on WiDr, SW620 and primary cultures treated by 5‐FU. Extracted proteins from different cell component (C for cytoskeleton; M for membranes; N for nucleus; Chr for chromatin; Ctsk for cytosquelet) were subjected to Western blot analysis as described in materials and methods section. Anti‐PARP antibody was used as a control for nuclear proteins, and anti‐Tubulin antibody was used as a control of cytoplasmic proteins. B, Indirect immunofluorescence staining was analysed using confocal microscopy (LSM 510 META; Zeiss, Göttingen, Germany). Anti‐GM130 was used as a Golgi marker and nuclei were stained with DAPI. Images were processed with the ZEN software application, and surface plots of the fluorescence data were generated with the image processing program ImageJ. C, ATF3 expression was analysed after subcellular fractionation of WiDr and SW620 as described just above

Because 5‐FU interferes with numerous cellular processes notably implicated in cytoskeleton organization and function^39^ and because sortilin is a key protein implicated in intracellular trafficking,^40^ its localization was evaluated in 5‐FU‐resistant cells (WiDr, SW620 and primary cultures). After 5‐FU long‐term treatment (one month), sortilin was found to be accumulated in membranes and in the cytoskeleton, contrary to control cells (Figure 3A). Moreover, sortilin localization is restricted to the Golgi as shown by its co‐localization with GM130, a Golgi marker (Figure 3B) which is more easily seen in SW620. Furthermore, transcriptomic regulation of sortilin expression was analysed in WiDr and SW620. Since it has been demonstrated that ATF3 is able to bind the *SORT1* promoter and repress *SORT1* transcription,^41^ ATF3 expression was assessed in cytoplasm and nucleus of the treated cells (Figure 3C). Our data showed that ATF3 expression strongly dropped after 5‐FU long‐term treatment, especially in nuclei where it exercises its transcriptional activity. So, the virtual disappearance of ATF3 could explain the observed overexpression of sortilin after 5‐FU‐prolonged treatment.

### 3.4 5‐FU treatment increases sortilin expression in tumours from Nude mice xenografted with CRC cell lines

**FIGURE 4:**
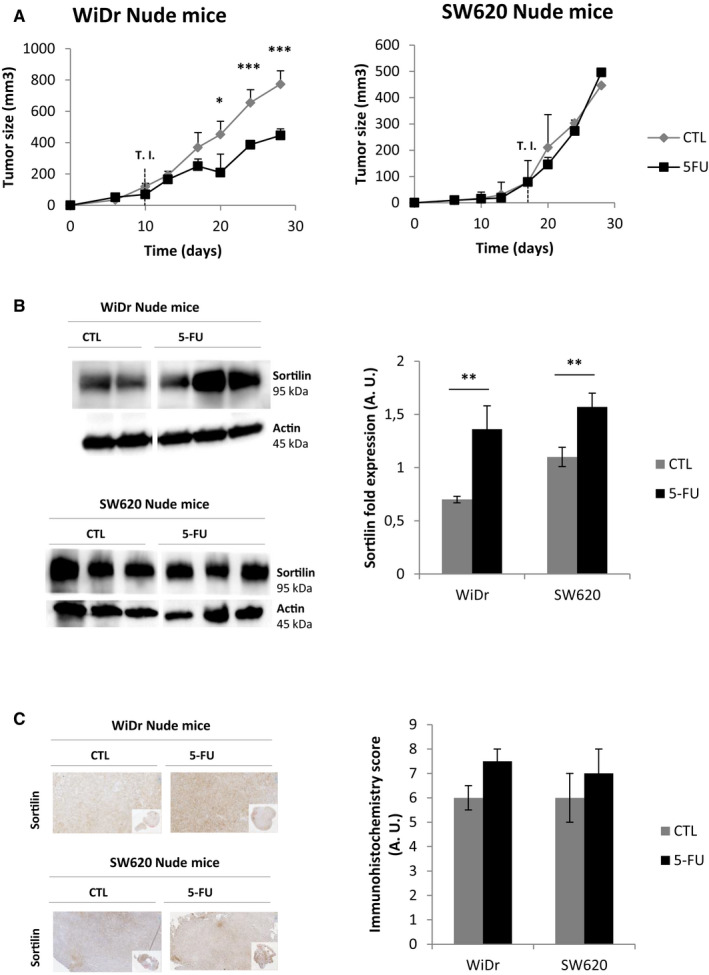
Sortilin protein expression increases upon 5‐FU treatment in tumours from Nude mice xenografted with CRC cell lines. A, Tumour volume of WiDr and SW620 xenografted Nude mice untreated (CTL) or exposed to 5‐FU (5‐FU) was determined every 3 days from the graft and during the treatment protocol, as described in material and methods (*V* = [L × W(L + W)]ᴨ/12). T. I. refers to Treatment Initiation. B, Sortilin protein expression analysis by Western blotting on whole lysates obtained from tumours of untreated xenografted Nude mice and 5‐FU‐treated ones (*n* = 5 per conditions). Actin was used as loading control. Histograms represent means of 5 different samples (significant *P*‐values are indicated in the graphs *P < .05, **P < .01, ***P < .001). C, Representative illustration of sortilin protein expression observed by immunohistochemistry (magnification x100) on paraffin‐embedded xenografted Nude mice tumours (*n* = 5 per conditions). Histograms represent the immunochemistry intensity score, as described in material and methods

In order to explore the correlation between sortilin expression and the responsiveness of tumours to 5‐FU, WiDr and SW620 were grafted into Nude mice. Once tumour volumes reached 100 mm^3^ (day 10 for WiDr and day 17 for SW620), mice were treated (Treatment Initiation, T. I.) with 5‐FU or vehicle (CTL) every 72 hours until sacrifice (day 28, ie one month). WiDr‐derived tumours regressed significantly relative to control after 15 days of 5‐FU treatment (Figure 4A). Despite the evident sensitivity of these tumours to 5‐FU, the regimen was not sufficient to induce total tumour regression, suggesting that a 5‐FU‐resistant subpopulation of cells survived the treatment. By contrast, SW620‐derived tumours were totally resistant to 5‐FU and did not regress at all (Figure 4A). Moreover, 5‐FU‐treated tumours from both WiDr and SW620 expressed elevated levels of the sortilin protein (Figure 4B,C) contrary to other NTs expressions (data not shown). In vivo, even though cells from low‐grade tumours (WiDr) responded better to 5‐FU than those derived from high grade tumours (SW620), sortilin protein expression was significantly higher in either one of the persistent tumours following 5‐FU treatment. These in vivo data are in complete agreement with the results obtained in vitro highlighting the role of sortilin as a marker of 5‐FU resistance.

### 3.5 SORT1 mRNA expression is associated with poor clinical outcomes and sortilin expression is positively correlated with CRC grades

**FIGURE 5:**
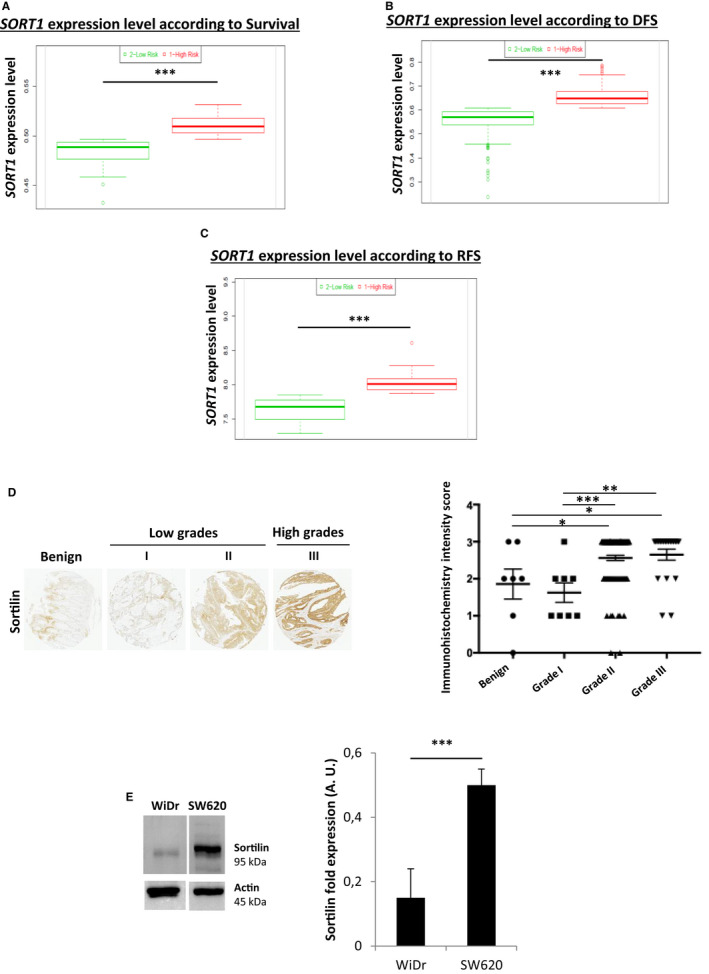
High *SORT1* expression is associated with poorer survival, DFS and RFS, and sortilin expression is associated with higher CRC tumour grades. *SORT1* expression levels by risk group according to survival (*n* = 77; A), DFS (*n* = 545; B) and RFS (*n* = 37; C) of patients suffering from CRC, extracted from ‘SurvExpress’ database. Significant P‐values are indicated in the graphs *P < .05, **P < .01, ***P < .001. D, Sortilin protein expression, observed by immunohistochemistry (magnification, 50×) on tissue microarrays including seven samples of benign tissue and 126 samples from CRC tumours of different grades (*n* = 8, 98, and 20 for grades I, II and III, respectively). Quantification was performed by calculating the immunochemistry intensity score described in materials and methods. E, Sortilin expression in whole‐cell lysates of WiDr and SW620. Actin was used as a loading control

Finally, once demonstrated the link between 5‐FU resistance and sortilin, we assessed its expression level in relation with prognosis of CRC. Thus, we checked the expression of *SORT1* mRNA in relation with patient status. The in silico analysis of data bases showed that *SORT1* mRNA expression levels were significantly elevated in high‐risk patient groups characterized by the worst survival, disease‐free survival (DFS) and relapse‐free survival (RFS), who are at the highest risk of developing new tumours after treatments (Figure 5A‐C). Moreover, ex vivo quantification of sortilin in tissues from 133 patients with benign (*n* = 7) or colorectal tumours from three tumour grades (*n* = 8, 98, and 20 for I, II, and III, respectively) revealed a positive correlation of sortilin protein expression with tumour grade (Figure 5D) while a simple upward trend was observed for CRC stages (data not shown). These observations have to be put together with those obtained in vitro: the sortilin level of the WiDr cell line, a reference for low tumour grade, was significantly lower than the level found in the SW620 cell line, a reference for high tumour grade (Figure 5E and Figure 1C). These findings about sortilin expression in various environments indicated that not only is sortilin a 5‐FU resistance marker but also a biomarker for CRC aggressiveness associated with poor clinical outcomes.

## 4 DISCUSSION

CRC is the second leading cause of cancer‐related deaths. First‐line chemotherapy of CRC uses 5‐FU, a pyrimidine analogue that inhibits thymidylate synthase, and whose metabolic activation results in its incorporation into RNA, which interferes with RNA function and prevents DNA synthesis. Even if 5‐FU remains the reference molecule used in combination with other chemotherapeutic drugs, it is highly toxic and can lead to patients' death.^42^ This observation recently suggested a fine monitoring^43^ implying assays of dihydropyrimidine dehydrogenase (DPD) activity,^44^, ^45^ the enzyme responsible for the catabolism of 5‐FU. Moreover, resistance to 5‐FU is a major clinical problem and its mechanism is highly complex.^7^ Even if different combinations are currently used to fight CRC, as referred as cytotoxic chemotherapy, molecular‐targeted therapy and immunotherapy, the need to find new and reliable biomarkers remains because resistance always comes soon or later and whatever combination used.^46^

Pathology tumour, node, metastasis (pTNM) staging, currently the most significant prognostic factor in CRC, is highly relevant to therapeutic decision‐making.^47^, ^48^ However, pTNM only reflects the anatomic extent of the tumour, and some patients suffering from stage II CRC, who are not eligible for post‐operative adjuvant chemotherapies, may experience outcomes similar to those of stage III CRC patients who receive adjuvant treatments. In light of this situation, other prognostic factors must be taken into account, such as tumour grade, to predict the clinical course of CRC, regardless of TNM stage.^47^ Tumour grading is based on histological differentiation state. Combined with tumour microsatellite instability (MSI) status, it is included in the histopathological assessment in CRC. Well and moderately differentiated grades are grouped as low grades, while poorly and undifferentiated grades are grouped as high grades that have poor prognosis if they exhibit micro satellite stability (MSS) but they behave as low‐grade tumours if they exhibit MSI.^48^, ^49^ However, identification of new biomarkers could improve prognostic tumour grading.^50^

In this context, the multifunctional protein sortilin represents a promising candidate even though its function is cell‐type–specific: in breast cancer^23^ and neuroendocrine tumours,^24^ it is associated with tumorigenesis and poor prognosis, but the opposite is true for non–small cell lung cancer.^12^ In this study, sortilin is overexpressed in vitro after 5‐FU treatments whether short (72 hours) or long (one month) and also in in vivo‐treated tumours. Elevated *SORT1* mRNA expression was associated with poorer survival, DFS and RFS in high‐risk CRC patients. Its overexpression is also associated with high CRC grades. These findings provide the first evidence that sortilin is a biomarker of 5‐FU resistance in CRC, which is associated with poorer prognosis and clinical outcomes, and higher tumour grades.

Consistent with this, the cell lines used in this study were chosen on the basis of their (a) tumour grades (low for WiDr; high for SW620^29^), (b) MSS status (MSS CRC patients are mostly eligible for 5‐FU), (c) contrasting mutational status (KRAS^WT^, BRAF^V600E^, PI3KCA^P449T^ and PTEN^WT^ for WiDr *versus* KRAS^G12V^, BRAF^WT^ and PI3KCA^WT^ for SW620) and (d) morphologic appearance linked to the consensus molecular subtype (CMS) classification (epithelial‐like phenotype, CMS3 for WiDr; mesenchymal‐like phenotype, CMS4 for SW620,^51^). On the other hand, primary cultures represent all tumour grades and also different mutational status at least for *KRAS* (as shown in Table 1). Overexpression of sortilin expression was observed in vivo in 5‐FU‐resistant tumours derived from both cell lines and in vitro (at the mRNA and protein levels) in whole‐cell lysates of 5‐FU‐resistant WiDr, SW620 and primary cultures obtained after short‐ (72 hours) or long‐term (one month) treatments. In addition, the drop in expression of ATF3, a down‐regulator of *SORT1* transcription, could explain sortilin overexpression.

However, clarifying the precise role of sortilin in chemotherapeutic resistance remains a challenge. The well‐known intrinsic functions of sortilin in the regulation of trafficking of NTs and their receptors^52^ do not seem to be responsible for 5‐FU resistance, owing to the levels of NTs receptors (TrkB and p75^NTR^) that, upon 5‐FU treatment, stay as elevated as those found in untreated cells. It thus seems that 5‐FU does not induce functional degradation and turnover of the NTs receptors. Consequently, classic pro‐survival signalling pathways implicated in carcinogenesis (PI3K/Akt and Erk1/2) are affected. Both Pi3K/Akt and Ras/Raf/mitogen‐activated protein kinases (MAPK) are inter‐connected pro‐survival pathways associated with cell proliferation and prevention of apoptosis,^53^, ^54^ which have been demonstrated to be involved in CRC cell 5‐FU resistance: Erk1/2 and PI3K/Akt pathways’ inhibition enhances 5‐FU cytotoxicity and apoptosis in CRC cells,^55^, ^56^, ^57^, ^58^ reinforcing the benefit of targeting proliferative pathways in association with chemotherapy. Even though no difference in *BRAF* or *KRAS* mutational status between 5‐FU‐resistant and ‐untreated CRC cells was observed (data not shown), analysis of other genes might be interesting. For example, *PTEN*‐activating and/or *PI3KCA*‐inhibitory mutations could explain the Akt inhibition observed in 5‐FU‐resistant CRC cells.^54^ Because Akt hyperactivation in pancreatic cancer cells results in subsequent inhibition of Raf and mitogen‐activated protein kinase kinase (MEK)/Erk1/2 pathway,^54^ and that MEK1/2 inhibition results in PI3K/Akt pathway activation in CRC cells,^56^ these two pathways take over from each other. In fact, the Akt activation observed in 5‐FU‐resistant CRC cells from low tumour grade cells (WiDr) could be consistent with the no significant Erk modification; and the Akt inhibition observed in 5‐FU‐resistant CRC cells from high tumour grade cells (SW620) could be consistent with increased Erk activation.

On another hand, 5‐FU disrupts numerous genes in CRC cell lines involved in nucleotide binding and nucleotide metabolism, mRNA processing, cytoskeletal organization, amino acid metabolism, signal transduction/transport and oxygen metabolism.^39^ This observation could explain our result on nuclear ATF3 inhibition, which acts as a transcriptional repressor^41^ on the *SORT1* promoter region. Moreover, 5‐FU effects on Golgi phosphoprotein 3 (Golph3), a Golgi membrane protein playing important role in Golgi trafficking and morphology, have been reported.^59^ These data could explain our results on sortilin accumulation as a consequence of 5‐FU effect on this organelle. However, further investigations of other cellular processes and proteins involved in drug resistance (eg multidrug resistance), detoxification proteins and reactive oxygen species^60^ might provide further insight into sortilin‐mediated 5‐FU resistance.

Finally, in patient analyses, sortilin constitutes a promising biomarker for aggressiveness and poor prognosis in CRC, associated with poor survival, DFS, and RFS. Moreover, even if more patients are needed, sortilin overexpression observed in high tumour grades from untreated patients suggests its possible incorporation into CRC tumour grading assessment.

Regardless of treatment duration, CRC tumour grade and whatever mutational status, sortilin seems to be a biomarker for 5‐FU resistance. In the context of personalized cancer therapy, monitoring sortilin expression could constitute a promising approach to (a) more accurately classify patients, in addition with other criteria, for improving the patient taking in care and to (b) explain 5‐FU resistance in CRC, for improving the knowledge of its mechanism. Finally, our work permits identifying a new potential therapeutic target and open the door to the research for finding new tools to fight against CRC.

## CONFLICT OF INTEREST

The authors declare that they have no conflict of interest.

## AUTHOR CONTRIBUTION

**Sabrina Blondy:** Conceptualization (equal); Formal analysis (equal); Methodology (equal); Writing‐original draft (equal). **Hugo Talbot:** Conceptualization (equal). **Sofiane Saada:** Conceptualization (equal). **Niki Christou:** Conceptualization (equal). **Serge Battu:** Supervision (equal); Validation (equal). **Julie Pannequin:** Writing‐review & editing (equal). **Marie‐Odile Jauberteau:** Writing‐review & editing (equal). **Fabrice Lalloué:** Writing‐review & editing (equal). **Mireille VERDIER:** Validation (equal); Writing‐original draft (equal); Writing‐review & editing (equal). **Muriel MATHONNET:** Validation (equal); Writing‐original draft (equal); Writing‐review & editing (equal). **Aurélie PERRAUD:** Conceptualization (equal); Supervision (equal); Validation (equal); Writing‐original draft (equal); Writing‐review & editing (equal).

## Supporting information

Fig S1

## Data Availability

Data sharing is not applicable to this article as no new data were created or analysed in this study.
