# Supplementary material for: Overexpression of sortilin is associated with 5‐FU resistance and poor prognosis in colorectal cancer
Source: J Cell Mol Med. 2020 Dec 16;25(1):47–60. doi: 10.1111/jcmm.15752 (PMC7810928; doi:10.1111/jcmm.15752)
Supplement: Supplementary file 1 — Fig S1 [file JCMM-25-47-s001.pdf]

Supplementary data

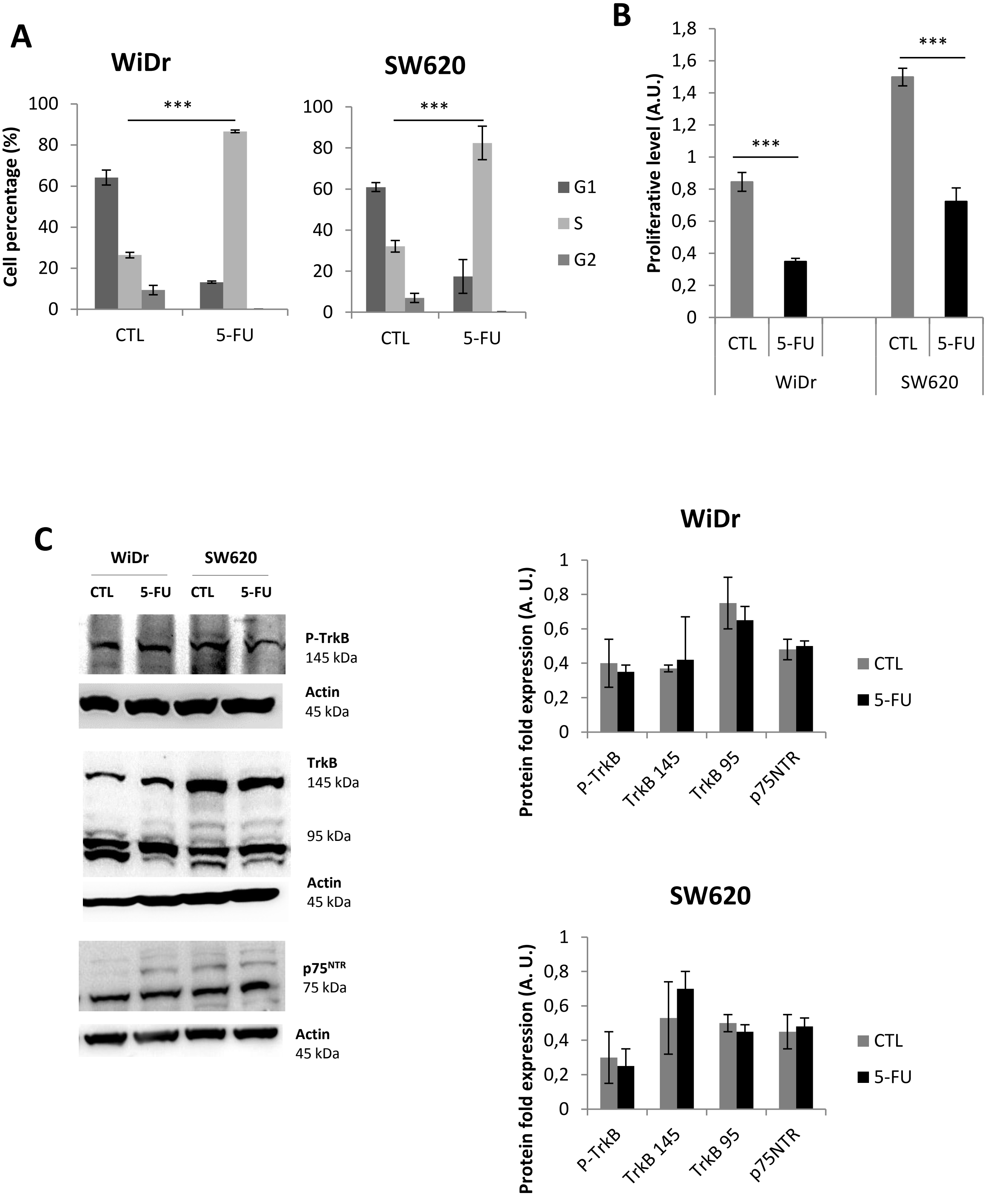

D

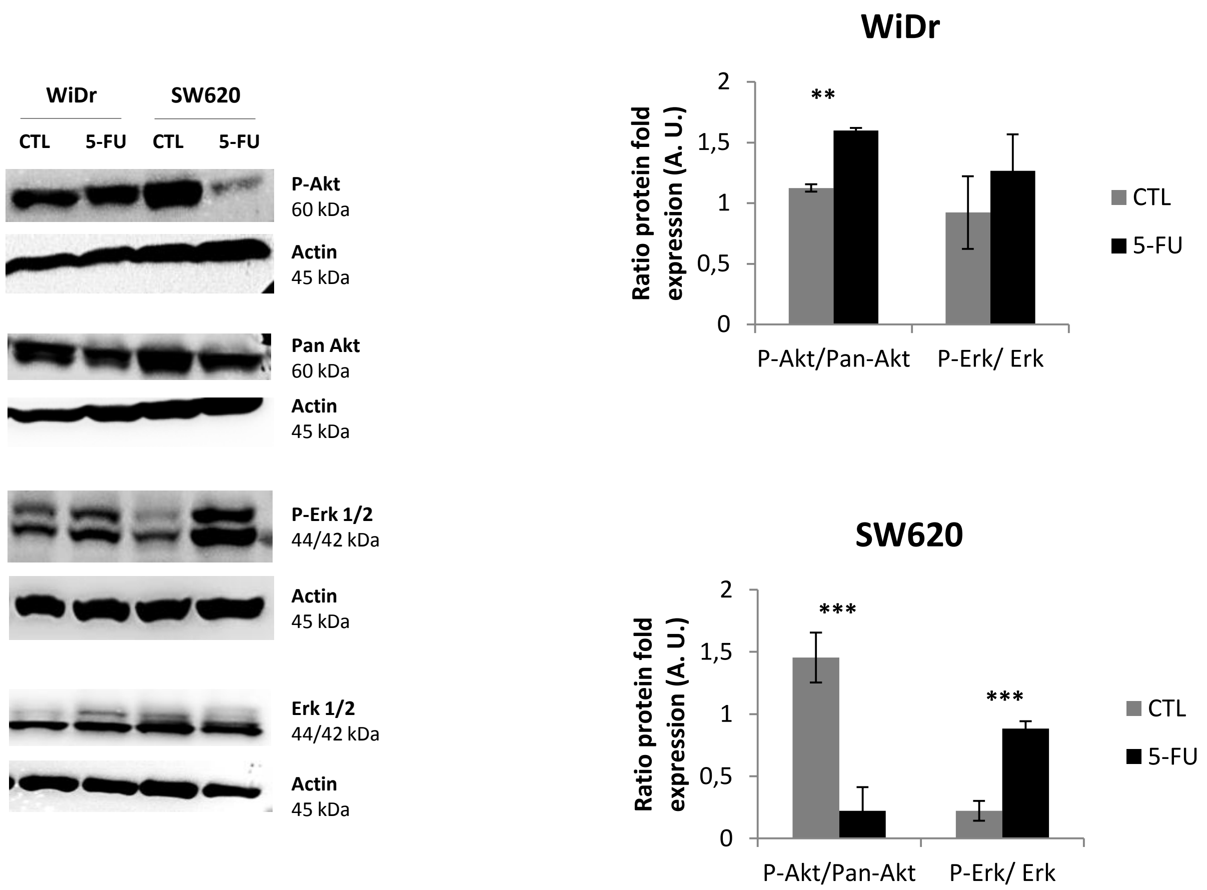

<u>Figure S1</u>: Short-term (8 µM, 72 h) 5-FU treatment consequences on cell cycle, proliferation and NTs pathways of WiDr and SW620 cells

WiDr and SW620 were treated with 8 µM 5-FU for 72 h and cell cycle **(A)** and proliferation rate **(B)** were determined as described in material and methods. **(C)** TrkB and p75^{NTR} protein expressions were analyzed by western blotting from whole cell lysates of 5-FU treated and untreated WiDr and SW620 cells (CTL). **(D)** P-Akt, Akt, P-Erk and Erk protein expressions were analyzed as previously described. The ratio P-Akt/Akt and P-Erk/Erk represents activation levels of both Erk and Akt. Actin was used as loading control. Histograms are the means from at least three independent experiments. Significant P-values are indicated in the graphs *P<0.05, **P<0.01, ***P<0.001.

Figure S1
